# Is There a Relationship Between Use of Anti-Vascular Endothelial Growth Factor Agents and Atrophic Changes in Age-Related Macular Degeneration Patients?

**DOI:** 10.4274/tjo.27448

**Published:** 2018-04-25

**Authors:** Süleyman Kaynak, Mahmut Kaya, Derya Kaya

**Affiliations:** 1 Dokuz Eylül University Faculty of Medicine, Department of Ophthalmology, İzmir, Turkey; 2 Dokuz Eylül University Faculty of Medicine, Department of Geriatric Medicine, İzmir, Turkey

**Keywords:** Anti-VEGF agents, geographic atrophy, age-related macular degeneration

## Abstract

Choroidal neovascularization due to age-related macular degeneration (AMD) is currently treated successfully with anti-vascular endothelial growth factor (VEGF) intravitreal agents. Emerging evidence suggests that anti-VEGF treatment may potentially increase development of geographic atrophy. However, there is not yet direct proof of a causal relationship between geographic atrophy and use of anti-VEGF agents in neovaskuler AMD. The aim of this review is to discuss the evidence concerning the association between anti-VEGF therapy and progression of geographic atrophy.

## Introduction

Intravitreal anti-vascular endothelial growth factor (VEGF) application has been the most effective treatment method in recent years for neovascular age-related macular degeneration (AMD).^1,2,3^ The common feature of the multicenter studies conducted in this area with different agents and for different purposes is that they first determined the efficacy and safety of these agents. In the MARINA and ANCHOR trials, monthly ranibizumab injections preserved visual acuity and maintained vision level, and this finding has been clearly demonstrated in evidence-based, controlled comparative studies.^1,2^ Two main points have recently been raised regarding the safety of anti-VEGFs. The first concern is local side effects such as endophthalmitis, vitreal hemorrhage, or retinal detachment, and the second is systemic side effects, especially cerebrovascular events. However, studies of these extremely rare adverse events showed that the use of these agents was not significantly associated with the likelihood of developing such complications.^1,2,3,4,5^

Retrospective analyses of multicenter studies have provided new and interesting findings. One example is evidence from the CATT^3^ trial which suggests a relationship between long-term anti-VEGF therapy and the development of geographic atrophy. The IVAN^4^ and HARBOR^6^ trials were also retrospectively analyzed in terms of this possible relationship and reported suspicious findings similar to those found in the CATT trial.^3,4,5,6,7,8^

Therefore, one of the most important questions of recent times is whether late geographic atrophy is really more prevalent in patients with long-term anti-VEGF use, and if so, what role the anti-VEGF agents play in the development of geographic atrophy. 

### Geographic Atrophy: Natural Course

Geographic atrophy is an age-associated pathology whose etiopathogenesis involves complex processes.^7,8,9^ The main factor is an atrophic process that begins in the retinal pigment epithelium (RPE) and choriocapillaris.^9^ Genetics and aging are the main risk factors.^10^ Parallel to senescence of retinal pigment epithelial cells, lipofuscin begins to accumulate in the cytoplasm due to slowing lysosomal activities, resulting in a vicious cycle. Metabolism slows with aging, especially lysosomal metabolism, and phagocytosed lipid-rich material does not dissolve, accumulating as a result. These deposits, particularly of lipofuscin, increase oxidative stress and accelerate aging. This vicious cycle leads to faster atrophy and RPE cell loss. Lipofuscin increases oxidative stress and RPE cell apoptosis.^11^ In geographic atrophy, autofluorescence imaging in particular shows RPE cells that are still viable but lipofuscin-laden concentrated along the margin of the advancing atrophic zone. After cell loss, this autofluorescence disappears and the area darkens, demonstrating RPE cell death. This phenomenon demonstrated by fundus autofluorescence can be assessed as the front of geographic atrophy expansion.^12^

In fact, geographic atrophy is atrophy of the RPE and choriocapillaris, and is consistent with the natural course of aging. In some patients, however, cells with an oncogenic phenotype undergo an exceptional change, with some regaining the ability to divide and starting to divide aggressively. In some patients who convert from geographic atrophic to wet AMD, the RPE cells exhibit high sensitivity to VEGFs, resulting in neovascularization. These appear as cases of wet AMD.^13^ In wet AMD patients, the neovascular process continues on one hand, while geographic atrophy continues as part of the natural disease course on the other hand. Therefore, while the underlying process of geographic atrophy continues in these wet AMD patients, they are also receiving intravitreal anti-VEGF therapy. In fact, geographic atrophy may be related to the ongoing natural course.^14^

### The Risk of Developing Geographic Atrophy due to Anti-VEGF Use: Results of Multicenter Studies

Significant visual gains can be achieved in AMD patients with choroidal neovascularization (CNV) with long-term intraocular injection of numerous anti-VEGF agents.^1,2,3,4,6^ However, there is debate in the literature regarding whether the geographic atrophy seen during long-term follow-up in these patients, who had received many anti-VEGF injections at high frequency, was a result of the natural course of the disease or was associated with the anti-VEGF molecules used. Our current understanding of the relationship between geographic atrophy and anti-VEGF use is summarized in Table 1.

It was noted with the CATT^15^ study that geographic atrophy may be associated with anti-VEGF agents. A retrospective evaluation of the CATT^15^ study revealed that geographic atrophy had developed in 18.3% of the patients (187 of 1024 patients) at the end of 2 years. It was also observed in the retrospective analysis that there was a difference between the monthly application and pro re nata (PRN) groups in terms of geographic atrophy. Of the patients who were administered monthly ranibizumab, 4.7% exhibited foveal atrophy and 21.1% extrafoveal atrophy at the end of year 2. These rates were 3.7% and 11.5%, respectively, in the patients who received ranibizumab PRN. Although the monthly and PRN ranibizumab groups did not differ significantly in terms of foveal atrophy development, the difference in extrafoveal atrophy rate was statistically significant. It was determined in the CATT^15^ study that the important common risk factors among patients who developed geographic atrophy were vision level of 0.1 or lower, retinal angiomatous proliferation, geographic atrophy in the fellow eye, and baseline intraretinal fluid. Conversely, factors associated with lower risk included blocked fluorescein, subretinal fluid thickness of 25 µm or more, subretinal tissue complex thickness of 275 µm or greater, and the presence of vitreoretinal adhesions. The CATT^15^ study compared the 1- and 2-year results of treatment with ranibizumab and bevacizumab. Although the patients in the ranibizumab group showed a higher risk of developing geographic atrophy, there was no difference in incidence between the groups at the end of the treatment regimen. Geographic atrophy was extrafoveal in the majority of patients. 

In contrast to the CATT, the 2-year results of the IVAN^4^ trial did not reveal a significant difference in geographic atrophy rates between patients treated with ranibizumab and those treated with bevacizumab (28% with ranibizumab, 31.2% with bevacizumab, p=0.46). When the results of the CATT^15^ and IVAN^4^ trial were interpreted together, the relationship between intravitreal agents and the development of geographic atrophy could not be proven definitively. However, the IVAN^4^ trial revealed a correlation between the development of geographical atrophy and the frequency of intravitreal anti-VEGF applications. At 2-year follow-up, the risk of developing geographic atrophy was reported as 34% with monthly intravitreal administration and 26% with PRN administration. The methods used to evaluate geographic atrophy in the CATT^15^ and IVAN^4^ studies were different. There was no agreement or consistency between the studies regarding the methodology of atrophy assessment. In the CATT^15^ trial, fundus fluorescein angiography (FFA) and color fundus imaging were used to detect atrophic areas. In the IVAN^4^ study, atrophic areas were visualized with FFA, color fundus, and optical coherence tomography (OCT) at baseline and during follow-up. Different techniques were also utilized to determine geographic area in the trials. However, there is still a lack of clarity concerning the questions of how geographic atrophy should be identified and which techniques (FFA, fundus autofluorescence, color fundus photography, OCT) should be used. The presence of active choroidal neovascular lesions presents the greatest challenge to the precise determination of the area of geographic atrophy. Atrophy is ideally detected by evaluating an atrophic area distant to the CNV lesion to demonstrate the effect of anti-VEGF therapy. The geographic atrophy surrounding areas of CNV may grow over time and merge with distant atrophic regions in the long term. Areas of geographic atrophy in CNV areas can actually be visualized with FFA and even with OCT, and their boundaries can be determined. 

In brief, despite different assessment techniques, both the 2-year results of CATT^15^ and the late subanalyses performed after conclusion of the IVAN^4^ trial showed that treatment was associated with higher incidence of geographic atrophy, but it was usually extrafoveal and did not affect vision significantly. They also indicated that the agents used were not influential in this phenomenon but that administration regimen may have an effect, with a PRN regimen being more favorable than monthly injections. Subanalysis of the HARBOR^6^ trial was similar to the CATT^15^ and IVAN^4^ trials. HARBOR^6^ is a Phase 3 trial in which the 2-year efficacy results of two different doses of ranibizumab (0.5 mg and 2 mg) with two different administration regimens (monthly/PRN) were evaluated in treatment-naive wet AMD patients with active subfoveal CNV (n=1097). Geographic atrophy was assesessed using FFA and color fundus images at 3, 12, and 24 months. Similar to the IVAN^4^ trial, baseline areas of atrophy were also taken into account in the HARBOR^6^ trial. Included in the areas of geographic atrophy were depigmented areas with prominent borders and increased visibility of choroidal vessels, areas with diameters greater than ≥250 µm, and attached, flat areas with prominent borders on FFA. However, atrophic areas with RPE tears were excluded. In the HARBOR^6^ trial, areas of atrophy adjacent to and nonadjacent to CNV were separately identified and evaluated. Lesions adjacent to CNV were especially included to achieve comparable results to the CATT^15^ and IVAN^4^ trials. In the HARBOR^6^ study, the incidence of atrophy in the eyes with no detectable atrophy at baseline was 29% according to results at 24 months. Based on this finding, there were no significant differences in atrophy incidence when compared with the CATT^15^ (20%) and IVAN^4^ (28%) trials. In the CATT^15^ trial, patients with baseline atrophy in the initial examination were not included in the evaluation. For this reason, the incidence of atrophy was found to be lower compared to the IVAN^4^ and HARBOR^6^ trials, which included patients with baseline atrophy. IVAN^4^ and HARBOR^6^ are more comparable in terms of patient groups, and the total incidence of atrophy, including existing (baseline) and newly developed atrophy, was equivalent at 28% and 29% respectively. In a subgroup analysis of the 5-year results of the CATT^16^ trial, the incidence of geographic atrophy was found to be 38%. The development of geographic atrophy was common and risk factors present at 2 years persisted at 5 years. The most important risk factors at start of treatment for the development of geographic atrophy were advanced age, poor visual acuity, widespread CNV, retinal angiomatous proliferation, geographic atrophy in the fellow eye, and intraretinal fluid. Thick subretinal tissue complex and presence of subretinal fluid were less associated with development of geographic atrophy. Incidence rates of geographic atrophy in post hoc analyses of the IVAN, CATT, and HARBOR trials are summarized in Table 2.

These findings point to two major conclusions from the HARBOR^6^ trial. One of these is that the agent used was not influential on the development of atrophy, as in the CATT^15^ and IVAN^4^ trials. In the HARBOR^6^ trial, it was observed that the dose (0.5 mg vs. 2 mg) and number (monthly vs. PRN) of ranibizumab injections administered were not associated with rates of atrophy development. 

Another important issue that must be considered in relation to geographic atrophy development is the effects of atrophic changes on visual acuity. Especially in the CATT^15^ trial, it may have been difficult to notice these extrafoveal atrophic areas if the retrospective analysis had not been performed, and since most of them had no effect on visual acuity, it is understandable that they could be overlooked by a researcher. In subanalysis of the study, no statistically significant difference was detected in the comparison of visual changes in patients with and without atrophy. 

## Conclusion

In conclusion, retrospective analyses of the CATT^15,16^, IVAN^4^, and HARBOR^6^ trials suggest that long-term intravitreal anti-VEGF therapies increase geographic atrophy in wet AMD patients. Even if this is the case, however, considering that 80% of these atrophic changes are extrafoveal and do not directly affect visual acuity, wet AMD patients should nevertheless be treated with adequate duration and frequency despite this possibility. As observed in the MARINA^1^ and ANCHOR^2^ trials, treatment yields visual gains of over 20 letters, compared to the loss of 14 letters in the sham group, which reflects the natural disease course. Even if atrophy does develop, the difference in letters gained between the patients with and without atrophy is 2.4 letters at 24 months. In light of these findings, it remains to be clarified whether the areas of geographic atrophy seen after anti-VEGF therapy in wet AMD are associated with the natural course of the disease or emerge as a result of the anti-VEGF molecules used in treatment. Regardless, considering the approximately 20-letter gain achieved over a 2-year period in these patients compared to the natural course, we believe these therapies are still indispensable for the treatment of wet AMD.

## Figures and Tables

**Table 1:**
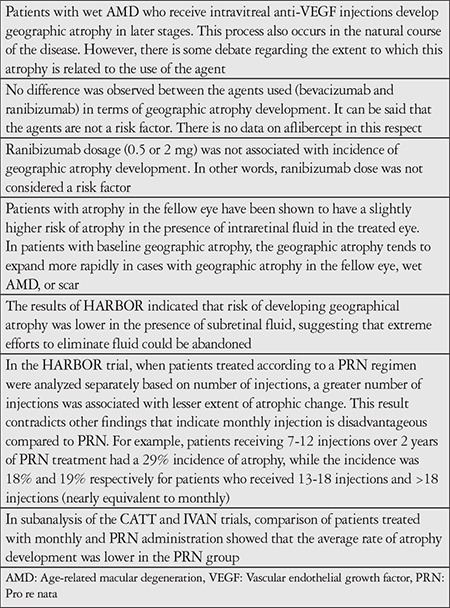
Evaluation of the relationship between geographic atrophy and anti-vascular endothelial growth factor use according to the literature

**Table 2:**
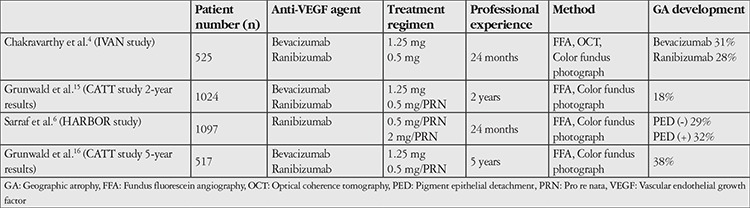
Comparison of the results of multicenter, randomized clinical trials showing the incidence of geographic atrophy related to anti- vascular endothelial growth factor use in wet age-related macular degeneration
